# Parasitic infection and oxidative status are associated and vary with breeding activity in the Seychelles warbler

**DOI:** 10.1098/rspb.2011.1865

**Published:** 2011-11-02

**Authors:** Janske van de Crommenacker, David S. Richardson, Amanda M. Koltz, Kimberly Hutchings, Jan Komdeur

**Affiliations:** 1 Behavioural Ecology and Self-organization, University of Groningen, PO Box 11103, 9700 CC, Groningen, The Netherlands; 2 Animal Ecology Group, Centre for Ecological and Evolutionary Studies, University of Groningen, PO Box 11103, 9700 CC, Groningen, The Netherlands; 3 Centre for Ecology, Evolution and Conservation, School of Biological Sciences, University of East Anglia, Norwich Research Park, Norwich NR4 7TJ, UK; 4 Nature Seychelles, PO Box 1310, Mahé, Republic of Seychelles; 5 Department of Biology, Duke University, PO Box 90338, Durham, NC 27708, USA

**Keywords:** parasitic infection, malaria, oxidative stress, reproduction, natural population, body condition

## Abstract

Parasites can have detrimental effects on host fitness, and infection typically results in the stimulation of the immune system. While defending against infection, the immune system generates toxic oxidants; if these are not sufficiently counteracted by the antioxidant system, a state of oxidative stress can occur. Here, we investigated the relationship between parasitic infection—using malarial infection as a model—and oxidative status in a natural population of the Seychelles warbler, while taking into account potentially interacting environmental covariates. We found that malaria is associated with increased susceptibility to oxidative stress, but this depends on the breeding stage: only during the energetically demanding provisioning stage did infected birds have higher oxidative stress susceptibility than non-infected birds. The imbalance in oxidative status was caused by a marked increase in oxidant levels observed only in infected birds during provisioning and by an overall reduction in antioxidant capacity observed in all birds across the breeding cycle. This finding implies that higher workload while dealing with an infection could aggravate oxidative repercussions. Malarial infection was not associated with body condition loss, suggesting that even when conditional effects are not directly visible, detrimental effects may still manifest themselves over the longer term through the oxidative consequences.

## 1. Introduction

Parasites and infectious diseases occur in nearly all ecosystems [1] and can have detrimental effects on the fitness of their host [2]. Being a powerful selection pressure in wild populations, parasites are considered an important selection force driving the generation and maintenance of evolutionarily important elements, such as genetic diversity and host life history [2,3]. In the battle against parasitic infection, host immune responses are central, but they also carry costs. For example, energetic investment for mounting an immune defence might be traded off with other nutrient-requiring functions, such as sexual signalling, breeding performance and growth [4–6]. The generation of oxidative stress is proposed to be another important cost of immune activation [7–9]. During an immune response, the generation of oxidants may take place via two routes: firstly, they are released by immune cells that use their cytotoxic effects to kill the pathogen [10]. Secondly, oxidants are by-products of oxygen consumption [11,12], and increased metabolic activity during an immune response [4,13] may contribute to the generation of additional toxic oxidants [11,14]. Yet, the link between metabolic activity and oxidant production is not straightforward, as animals can use alternative pathways for mitochondrial respiration to reduce the generation of oxidants [15,16]. Thirdly, parasites themselves can also be directly responsible for oxidant release through degradation products of their own metabolism [17,18].

While useful in immune protection, non-targeting toxic oxidants have a potentially important negative side-effect: they may damage host tissues [19] and may obstruct their functioning [20]. Accumulation of oxidative damage may eventually lead to degenerative pathologies, accelerated senescence and shortened lifespan [11]. Organisms minimize these harmful effects through antioxidant defences, which comprise an arsenal of endogenous and exogenous compounds that impede oxidant chain reactions by directly neutralizing the oxidants [10]. An imbalance in favour of oxidants over antioxidants represents the oxidative stress level [11,21].

Oxidative status is an important physiological index to consider when investigating associations between infection and host health status: besides the aforementioned potential direct link to immune function [9,22], it has a potential role as mediator of life-history trade-offs [23]. An important fitness-related strategy could be maternal antioxidant investment into offspring (e.g. antioxidants in eggs) in order to enhance offspring quality ([24]; reviewed in Surai *et al.*[25] and Blount *et al*. [26]).

Here, we study variation in oxidative status in wild animals exposed to parasites, using malaria as a model for parasitic infection. Malaria is an important protozoan parasite in a variety of mammals, reptiles and birds, and is transmitted from host to host via biting insects, such as mosquitoes [27]. Avian malaria, caused by parasites of the genera *Leucocytozoon*, *Plasmodium* and *Haemoproteus*, has been recorded in many bird species [2,28]. In captive birds, avian malaria has been shown to have pathogenic effects on the host, causing loss of energy, reduced growth and a stimulation of immune responses [29,30]. Also in natural systems, malarial infection is generally characterized by the stimulation of immune responses [31–33], as well as by reductions in body condition and parental care [34]. These types of effects are thought to lead to the loss of fitness observed in infected wild hosts [34–38], but see [39,40]. The impact of parasitic (malarial) infection on oxidative status is, as yet, nearly unexplored in ecological studies. Malarial parasites in wild-living hosts normally manifest themselves as latent infections with relapses during stressful situations such as breeding [28,41]. Therefore, it is important to determine whether stressful situations (e.g. heightened breeding activity) interact with the physiological impact of malarial infection and whether these links covary with other factors that are experienced by the individual.

We examine the links between malarial infection, oxidative status and body condition in the Seychelles warbler (*Acrocephalus sechellensis*), a passerine endemic to the Seychelles Islands. Moreover, we examine the extent to which these associations interact with individual characteristics, life-history stages and environmental circumstances. The population of Seychelles warblers on Cousin Island has been monitored intensively throughout breeding attempts since 1985. There is nearly no migration on or off the island [42], therefore, the population provides a closed study system. In this population, the GRW1 *Haemoproteus* strain of avian malaria [43] has been detected in *ca* 40 per cent of individuals, whereas no other parasites (either in the blood or in the gastrointestinal tract) have been detected [44,45]. The simplified nature of this parasite community, a relatively common feature of isolated island populations [46,47], greatly facilitates the analyses and interpretation of relationships between parasite infection and physiological status in comparison with species where complex multiple parasite communities are present.

We screened individual Seychelles warblers for avian malaria and assessed oxidative balance using the same (split) blood sample. For the oxidative balance, we measured both the oxidant component (measured as reactive oxygen metabolites, ROMs) and the antioxidant component (OXY). Malarial infection is expected to generate ROMs through activation of the immune system and possibly also through its own metabolism. Differences between individuals (e.g. sex, age and social status), their environmental circumstances (e.g. habitat quality) or their stage within the breeding cycle may all play important roles in the strength of the immune activation and its oxidative aftermath [9,48], so it is important to test interactions with these variables in the models. By investigating the relationships of these variables in a wild system, this study will provide a valuable contribution to our understanding of how parasitic infection affects individuals under natural circumstances.

## 2. Material and methods

### (a) Study population and data collection

Data were collected from the Seychelles warbler population on Cousin Island (29 ha; 04°20′ S, 55°40′ E), during the main breeding seasons (July–September) of each year in the period 2006–2008. The population has been studied intensively since 1985 [49–51]. From 1997 onwards, almost every individual has been individually ringed and monitored and consequently, the life history of nearly all individuals is known [50,52,53]. The Seychelles warbler population on Cousin is a cooperatively breeding system [54] with group sizes that range from two to six individuals [55,56], and birds inhabit year-round stable territories with the same mate, where they often remain throughout their lives [54].

Birds were caught using mist nets that were checked at least every 15 min. This study focused on adult birds only (i.e. birds older than eight months; [57]), as growing juveniles may have incomplete antioxidant machinery [58,59] or an immune system still in a developmental stage [60,61]. For each captured bird, a series of potentially relevant variables were identified: (i) Social status: based on field observations. Within a territory, the ‘primary’ male and female were defined as the dominant birds. All other resident adult birds were defined as ‘subordinates’ [62], which could either be ‘helpers’ (that assist in the care of young) or ‘non-helpers’ (that do not assist). Social status may be important, as it could reflect differences in individual quality or immunocompetence (predicting the direction is tricky: immunocompetence might be highest in dominants because they may have a better body condition [63–65], or weaker because they may invest more energy into reproduction (and associated hormonal elevations [66,67]) than subordinates). (ii) Breeding activity: each territory was checked for nesting activity at least once every two weeks by following the dominant female for 15 min [54]. Active nests were monitored throughout the breeding season to ascertain breeding stage (three categorical stages: pre-nesting, nest care (from the start of nest-building until hatching of the young) and provisioning (from the hatching of the young until their independence)). (iii) Territory quality: based on insect prey availability [54,68] that has been shown to be associated with oxidative status ([69]; see explanation regarding the calculation of territory quality). (iv) Sex (see below). (v) Age (in years): based on the long-term ringing data. (vi) Group size: the number of adults present in the territory. (vii) Time of day (minutes since sunrise at 6.00 h).

For each bird, mass (±0.1 g) and tarsus length (±0.1 mm) were measured. A blood sample (*ca* 100 µl) was collected immediately by brachial venipuncture using heparanized capillary tubes. Part of each sample (*ca* 20 µl) was diluted in 1 ml of 100 per cent ethanol in a screw-cap microfuge tube and stored at room temperature. This sample was used for malaria screening. DNA was extracted (following [52]) and sex was confirmed using the molecular (polymerase chain reaction, PCR) sexing method devised by Griffiths *et al*. [70]. This molecular analysis of sex was also used as a check to confirm that the extracted DNA samples were suitable for PCR amplification before proceeding with the malaria screening. The remaining blood (*ca* 80 µl) was centrifuged at 8000 r.p.m. for 8 min within 3 h of bleeding. The obtained plasma was stored frozen (−18°C) on average for 67 ± 22 days (minimum 27 days and maximum 119 days) until the analyses of oxidative status. Possible effects of storage time and method on oxidative parameters were tested (see below). Full details regarding the storage and transport of samples are provided in van de Crommenacker *et al*. [69].

### (b) Malaria screening

Malaria infection was screened for by using a nested PCR technique [71]. Molecular techniques have been found to be more accurate for malaria screening than the traditional blood smears [72,73], and they allow identification of the species/lineage by sequence information [74,75]. The method consisted of 20 cycles using the primers HaemNF1 and HaemNR3, followed by a final amplification of 35 cycles using the primers HaemF and HaemR2, which target a 479 bp section of the cytochrome b gene of *Haemoproteus* and *Plasmodium*. The genetic lineage of malaria was determined by sequencing positive amplicons using Big Dye terminator kit v. 3.1 (Applied Biosystems) following Hutchings [44]. The sequencing reaction was as follows; 1 µl Big Dye, 1.5 µl sequencing buffer, 0.5 µl HaemF or HaemR2 primer (10 µM), 5 µl H_2_O and 2 µl template. The thermal profile was 25 cycles of 96°C for 10 s, 50°C for 5 s and 60°C for 4 min. Sequences were run on a ABI3730 sequencer (Applied Biosystems), aligned using MEGA [76] and then compared with previously detected sequences in the National Centre for Biotechnology (NCBI) gene bank database using basic local alignment search tool (BLAST).

### (c) Measurement of oxidative status

Oxidative parameters in the blood plasma were measured with the d-ROMs and OXY-Adsorbent assays (Diacron, Grosseto, Italy). The d-ROMs assay measures the plasma concentration of hydroperoxides, a group of ROMs that derive from oxidative damage to biomolecules early in the oxidative cascade. Antioxidant capacity (OXY) was analysed using the OXY-Adsorbent assay, which quantifies the contribution of a large section of exogenously and (non-enzymatic) endogenously synthesized antioxidants. The capacity of the plasma antioxidants to neutralize oxidants was tested by adding a highly potent oxidant, hypochlorous acid (HClO). The unreacted radicals of HClO react with a chromogenic substrate, and the coloured complex that develops was measured photometrically. The concentration of this coloured complex is directly proportional to the concentration of HClO and indirectly related to the antioxidant capacity. Instructions provided with the kits were followed with some modifications (ROMs (in endpoint mode): volumes: buffer 400 µl, chromogen 4 µl, calibrator 10 µl, plasma sample 20 µl. Incubation: 90 min at 37°C; OXY: volumes: buffer 500 µl, chromogen 10 µl, calibrator 5 µl and sample 10 µl. Dilutions : calibrator 1 : 200, sample 1 : 50; incubation: 10 min at 37°C). Absorbencies were measured at 505 nm (spectrophotometer DU-720, Beckman Coulter, Woerden, Netherlands). ROMs are presented as millimolar of H_2_O_2_ equivalents, and OXY as millimolar HClO neutralized. Inter-assay variation was 2.25 per cent (ROMs) and 1.85 per cent (OXY), and intra-assay variation was 1.68 per cent (ROMs) and 3.08 per cent (OXY). A detailed description of the protocols can be found in Costantini & Dell'Omo [77].

### (d) Data analyses

**Figure 1.**
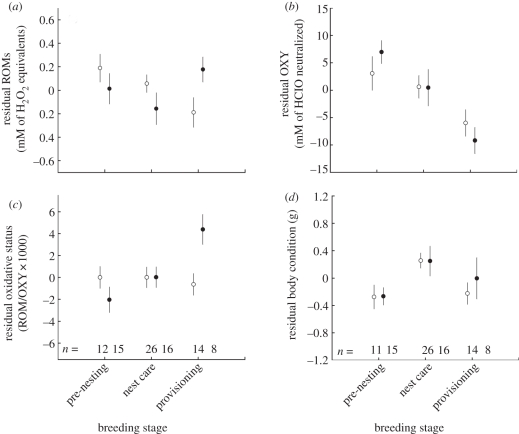
(*a*) ROMs, (*b*) OXY, (*c*) oxidative status, and (*d*) body condition throughout the breeding season in malaria-infected and non-infected Seychelles warblers. To correct for variation caused by variables other than malarial infection or breeding stage, residuals from the final model of electronic supplementary material, table S1 (excluding the variables ‘malaria’ and ‘breeding stage’) were used. Dots indicate mean ±s.e. and numbers indicate sample sizes. (*a*–*d*) Open circles, non-infected; filled circles, infected.

**Figure 2.**
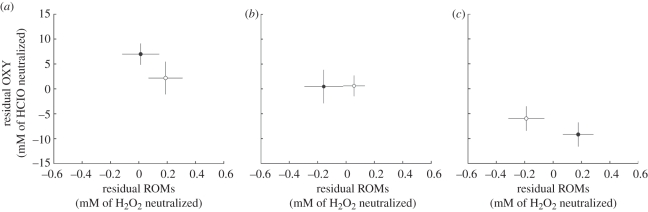
Relationships between ROMs and antioxidant capacity (OXY) during: (*a*) pre-nesting, (*b*) nest care and (*c*) provisioning in malaria-infected and non-infected Seychelles warblers. ROMs and OXY are plotted as residuals from the final models of electronic supplementary material, table S1 (excluding the variables ‘malaria’ and ‘breeding stage’). Dots indicate group means ±s.e. (*a*–*c*) Open circles, non-infected; filled circles, infected.

All observations were on different birds, i.e. no repeated measures on the same individuals. A mixed-modelling procedure in the programme MLWiN v. 2.20 [78] was used, with territory identity (*n* = 59) included as random effect. In MLWiN, variance at the individual level represents ‘residual variance’, and therefore individual identity was also included as random effect. To investigate links between malarial infection and the oxidative indices, models were constructed with either ROMs, OXY or the ratio between the two (as a measure of oxidative status and likely to indicate oxidative stress susceptibility, calculated as ROM/OXY × 1000 [79]) as the dependent variable (table 1*a*–*c*; electronic supplementary material, table S1*a*–*c*) and the following explanatory variables: malarial infection (infected or not), social status (dominant, helper and non-helper), breeding stage (pre-nesting, nest care and provisioning), field season, sex, territory quality, time of day and size-corrected body mass (residual of body mass × tarsus length; a commonly used indicator of body condition [80]; reviewed in Green [81]), group size and age (range 1–14 years). Territory quality data were log-transformed after which they approximated a normal distribution. Second-order polynomial functions of time of day and age were added to the models to allow for quadratic relationships. For figures 1 and 2, residuals from the final model of the electronic supplementary material, table S1 (excluding the variables ‘malaria’ and ‘breeding stage’) were used, in order to correct for variation caused by factors other than malarial infection or breeding stage.

**Table 1.** Model summary examining associations between malarial infection and: (*a*) ROMs, (*b*) OXY, (*c*) oxidative status, and (*d*) body condition in adult Seychelles warblers. The table shows malaria-related variables as left in the final model. The complete model that includes all other variables described in the methods section, can be found in the electronic supplementary material. Bold text indicates significance.

interaction	d.f*.*	(*a*) ROMs			(*b*) OXY			(*c*) oxidative status (ratio)			(*d*) body condition		
		estimate ± s.e.	*X*^2^	*p*	estimate ± s.e.	*X*^2^	*p*	estimate ± s.e.	*X*^2^	*p*	estimate ± s.e.	*X*^2^	*p*
**malaria**^**a**^	1	−0.14 ± 0.19	0.54	0.46	−2.54 ± 4.83	0.28	0.6	−2.09 ± 1.70	1.52	0.22	0.13 ± 0.16	0.60	0.44
**breeding stage**^**b**^	2		**6.34**	**0.042**		**30.40**	**< 0.001**		0.28	0.87		4.32	0.12
nest care		−0.19 ± 0.17			−16.19 ± 5.43			0.81 ± 1.57			0.45 ± 0.22		
provisioning		−0.47 ± 0.20			−39.43 ± 6.75			0.54 ± 1.85			0.21 ± 0.25		
**malaria**^**a**^**× breeding stage**^**b**^	2		**7.21**	**0.027**		3.13	0.21		**9.23**	**0.01**		0.58	0.75
infected × nest care		−0.14 ± 0.25			−14.74 ± 10.95			2.02 ± 2.13			0.27 ± 0.37		
infected × provisioning		0.51 ± 0.26			−21.58 ± 12.21			7.09 ± 2.42			0.21 ± 0.40		

^a^‘Non-infected' reference category.

^b^‘Pre-nesting stage' reference category.

To verify the robustness of the oxidative status ratio calculation, the analysis was repeated using a (similar two-level structured) model with ROMs as the dependent variable and OXY included as a covariate [69,82] along with all variables present in the final models shown in the electronic supplementary material, table S1. Furthermore, ROMs and OXY were integrated into one figure (figure 2) to show how ROMS relate to OXY in each breeding stage (pre-nesting, nest care and provisioning) in malaria-infected and non-infected Seychelles warblers. This way, the variability in both ROMs and OXY is visible. The non-significant correlation between residual ROMs and OXY (ß = −0.004 ± 0.004, 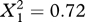, *p* = 0.40; and with raw data: *p* = 0.20) indicated that ROMs and OXY varied independently.

The model investigating the association between malarial infection and body condition (table 1*d*, electronic supplementary material, table S1*d*) was constructed with body mass as the dependent factor and with tarsus length included as a covariate to correct for structural size differences between individuals. All biologically relevant interactions were tested but only reported when statistically significant. Model selection was based on the step-wise exclusion of non-significant terms in the order of their significance assessed by their Wald statistic. Significance level was set at *p* < 0.05. Significance levels for post hoc tests were adjusted for multiple comparisons using the false discovery rate (FDR) procedure [83].

## 3. Results

Malarial infection was associated with both ROMs (table 1*a*) and the oxidative status ratio (table 1*c*). Both of these relationships were present as an interaction effect with breeding stage (ROMs: *p* = 0.027, table 1 and figure 1*a*, oxidative status ratio: *p* = 0.01, table 1 and figure 1*c*). The latter result was also found when analysing oxidative status by taking ROMs as dependent variable and including OXY as a covariate (*p* = 0.009). Malarial infection was not associated with OXY or body condition (table 1, both *p* > 0.44).

Post hoc investigation of the malaria × breeding stage interaction (tables 2 and 3) revealed that this was based on differences between non-infected and infected birds during the provisioning stage (figure 1). In the first two stages of the breeding cycle (pre-nesting and nest care), ROMs were not significantly different for non-infected and infected birds (*p* > 0.09; figure 1*a*) and did not change between the two stages (non-infected: *p* = 0.24 and infected: *p* = 0.13; figure 1*a*). Then, during provisioning, ROMs in non-infected individuals decreased to levels lower than measured during pre-nesting (*p* = 0.016) and nest care (*p* = 0.05). By contrast, ROMs in infected individuals tended to increase to levels higher than measured during nest care (*p* = 0.08). This resulted in significantly higher ROMs in infected individuals compared with non-infected individuals during provisioning (*p* = 0.05, figure 1*a*).

**Table 2.** Post hoc tests: differences in oxidative parameters between non-infected and infected adult Seychelles warblers during each breeding stage. Significant *p*-values are shown in bold.

interaction malaria × breeding stage	(*a*) pre-nesting			(*b*) nest care			(*c*) provisioning		
	estimate ± s.e.	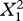	*p*	estimate ± s.e.	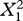	*p*	estimate ± s.e.	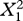	*p*
effect of malaria^a^ in:									
ROMs	−0.14 ± 0.19	0.54	0.46	−0.24 ± 0.14	2.89	0.09	**0.37 ± 0.19**	**3.84**	**0.05**
OXY	11.08 ± 9.01	1.51	0.22	−3.62 ± 6.40	0.32	0.57	−10.44 ± 7.57	1.90	0.17
oxidative status (ratio)	−2.09 ± 1.70	1.52	0.22	−0.07 ± 1.31	0.003	0.96	**5.00 ± 1.77**	**7.95**	**0.005**
body condition	−0.02 ± 0.26	0.01	0.92	0.24 ± 0.27	0.780	0.38	0.19 ± 0.31	0.38	0.54

^a^Reference category is ‘non-infected’.

**Table 3.** Post hoc tests: patterns of oxidative parameters and body condition throughout the breeding season in non-infected and infected Seychelles warblers. Significant *p*-values are shown in bold.

	interaction malaria × breeding stage	(*a*) non-infected			(*b*) infected		
		estimate ±s.e.	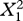	*p*	estimate ±s.e.	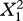	*p*
ROMs	pre-nesting^a^ and nest care	−0.19 ± 0.17	1.36	0.24	0.30 ± 0.20	2.32	0.13
	pre-nesting^a^ and provisioning	**−0.47 ± 0.20**	**5.82**	**0.016**	0.04 ± 0.22	0.03	0.87
	nest care^a^ and provisioning	**−0.28 ± 0.14**	**3.77**	**0.05**	0.34 ± 0.19	3.03	0.08
OXY	pre-nesting^a^ and nest care	−8.64 ± 7.31	1.40	0.24	**−23.35 ± 8.05**	**8.42**	**0.004**
	pre-nesting^a^and provisioning	**−29.56 ± 8.96**	**10.89**	**<0.001**	**−51.08 ± 9.41**	**29.45**	**<0.001**
	nest care^a^ and provisioning	**−20.92 ± 6.25**	**11.20**	**<0.001**	**−27.74 ± 7.66**	**13.12**	**<0.001**
oxidative status (ratio)	pre-nesting^a^and nest care	0.81 ± 1.57	0.26	0.61	2.83 ± 1.63	3.03	0.08
	pre-nesting^a^ and provisioning	0.54 ± 1.85	0.08	0.77	**7.62 ± 1.96**	**15.16**	**<0.001**
	nest care^a^and provisioning	−0.27 ± 1.33	0.04	0.84	**4.79 ± 1.78**	**7.29**	**0.007**
body condition	pre-nesting^a^ and nest care	0.46 ± 0.32	2.10	0.15	**0.73 ± 0.27**	**7.10**	**0.008**
	pre-nesting^a^ and provisioning	0.29 ± 0.38	0.57	0.45	0.50 ± 0.36	1.93	0.17
	nest care^a^ and provisioning	−0.13 ± 0.40	0.11	0.74	−0.18 ± 0.39	0.33	0.57

^a^Reference category for post hoc comparison.

For OXY, no malaria-related interaction was found, but there was a strong reduction in OXY throughout the breeding season in both infected and non-infected individuals (tables 1 and 3; figure 1*b*).

During provisioning, both models investigating oxidative status indicated that infected individuals had a significantly higher oxidative imbalance (higher oxidative stress susceptibility) than non-infected individuals as a consequence of the high ROM levels and the low OXY (oxidative status ratio: *p* = 0.005; ROMs with OXY as a covariate: *p* = 0.022, figure 1*c*). This oxidative imbalance in infected individuals was significantly greater during provisioning than during both earlier breeding stages (oxidative status ratio: compared with pre-nesting, *p* < 0.001 and compared with nest care, *p* = 0.007; ROMs with OXY as a covariate: compared with pre-nesting, *p* = 0.023 and compared with nest care, *p* = 0.041). Figure 2 shows that only during provisioning, ROMs were higher in infected birds. Furthermore, there was no rise in OXY, indicating a state of oxidative imbalance (or, increased oxidative stress susceptibility).

Body condition did not differ between non-infected and infected birds in any of the breeding stages, nor did it differ between the provisioning stage and the earlier stages in any group. Overall samples, body condition increased during nest care, but returned to pre-breeding values during the provisioning stage (figure 1*d*).

Possible effects of storage time and method on oxidative parameters were found to be non-significant (time period between bleeding and centrifuging (range 3–182 min); ROMs: *p* = 0.92, OXY: *p* = 0.88; cooling method during this time period (i.e. fridge versus cool box); ROMs: *p* = 0.25, OXY: *p* = 0.67; time period between sample collection and assaying (range 27–119 days); ROMs: *p* = 0.33, OXY: *p* = 0.42).

## 4. Discussion

In the Seychelles warbler, we found an association between parasitic (malarial) infection and oxidative status that varied with breeding stage. Infected birds had significantly higher oxidative imbalance (i.e. oxidative stress susceptibility) than non-infected birds, but this occurred only during the provisioning stage of breeding and not during the pre-nesting or incubation stages. This imbalance was the result of ROMs being significantly higher in infected than uninfected provisioning birds, whereas OXY was relatively low in all birds compared with earlier breeding stages.

There are two main reasons why ROMs may be higher in infected birds during provisioning. Firstly, this stage of the avian breeding cycle is the period in which energetic demands and resource constraints are assumed to be greatest [84,85]. The heightened physical activity (and associated metabolic consequences) involved with the feeding flights [86,87] is expected to stimulate the generation of oxidants ([88]; but see [15,16]). However, we found elevated ROMs only in infected birds. In these birds, ROMs may be elevated for additional reasons. Firstly, immune activation may have been triggered by the malarial infection. Oxidant-producing cellular immune responses are known to be involved in the control of malarial infection [31]. Secondly, in infected birds, ROMs are also produced as a direct result of the metabolism of the parasite itself. During its life stages within the hosts' red blood cells, the malaria parasite degrades haemoglobin and uses the amino acids gained for its own growth [17,18]. The degradation products (haeme monomers) formed are capable of generating toxic oxidants [18,89]. The occurrence of such a direct oxidative effect has been confirmed for *Haemoproteus* [18], the genus of malaria parasite present in the Seychelles warbler [44,45]. However, it is well possible that these parasite-generated oxidant concentrations are too low to be picked up by the ROMs assay.

OXY levels during the provisioning stage were low in both non-infected and infected birds, which may be due to the decline of internally stored antioxidants [90,91]. To deal with variable environmental conditions and antioxidant protection demands, birds may build up a ‘buffer’ of stored (dietary) antioxidants that can be mobilized whenever needed ([90,91]; reviewed in Monaghan *et al*. [23]). Maintenance of this internal antioxidant store is thought to be easy when dietary antioxidants are abundant and resource needs for other functions are low. However, during breeding, foraging time to obtain food (and thus dietary antioxidants) for their own needs is limited, and resources may also be used in reproduction-related functions, such as sexual signalling [92,93] and antioxidant allocation to eggs [26,94]. As a consequence, antioxidant stores may be exhausted over the course of the breeding season at the expense of the buffer that is normally available for oxidative damage protection. Owing to these relatively low levels of OXY during the later stages of breeding (provisioning), individuals are even more susceptible to any elevations in ROMs that occur and—without sufficient counteraction—might enter a state of oxidative stress (figures 1*c* and 2*c*; note that oxidative status results obtained by the calculation as a ratio between ROMs and OXY were confirmed by results obtained from the model with ROMs as the dependent variable and OXY as a covariate).

Unlike for the oxidative parameters, there was no association between malarial infection and body condition in the present study. We would have expected infected individuals to be leaner than uninfected ones owing to exhaustion of body reserves while combating the infection. However, we found no evidence that the detrimental effects of infection were externally visible through condition loss. A lack of relationship (or even a positive relationship [31]) between infection and condition has been reported in previous studies ([95,96]; but see [34,97]). Our results suggest that parasitic infection could have longer term detrimental effects through its oxidative repercussions. While not directly externally visible, this may accelerate the degeneration of body functions over time [11]. Obviously, testing and confirming such speculation are beyond the possibilities of our correlative study. Nevertheless, our study highlights that the physiological consequences of disease can be complex, and effects on health status may extend beyond individual physiological systems (i.e. mass loss and immune activation) [14,98,99].

As our study was based on single samples of different birds, we cannot totally rule out the possibility that the different breeding stages in our dataset were represented by individuals of different quality (e.g. relatively better quality birds present in the later stages of reproduction). Consequently, the observed effects of malarial infection during the provisioning stage could still be confounded by misjudged individual quality, e.g. if the provisioning stage mainly comprises high-quality individuals that were able to mount a stronger immune response, the oxidative consequences of parasitic infection during this stage may have been overestimated. However, we have evidence that the birds in the provisioning stage are not biased with regards to oxidative status. First, we found no relationship between body condition and breeding stage. Second, breeding success is found not to be linked with oxidative status in this species [100]. Third, malarial infection is not related with either reproductive success or adult survival in this species [44,45], which implies that malarial infection does not contribute to birds deserting the nest or dying (while nesting). This evidence suggests that there is no confounding effect, but a follow-up study based on repeated samples of birds in the different breeding stages would be needed to fully exclude the possibility of such bias.

Parasitic infection-related variation in oxidative status was not linked with social status. Birds with different social status may differ in individual quality and their immunocompetence. However, although we found status-related differences in body condition (the three-way interaction between status, sex and breeding stage, electronic supplementary material, table S1, and see [63]), we did not find any evidence that social status was linked to infection-related differences in oxidative balance. Furthermore, as Seychelles warblers with different social status differ in their contribution to the breeding process [62], we tested for the presence of a three-way interaction effect between malaria, breeding stage and social status, but this was non-significant for all of the dependent variables (all *p* > 0.27).

Further research is needed to disentangle the extent to which elevated ROMs (and consequently oxidative stress susceptibility) in malaria-infected provisioning birds are derived from the increased workload and from activation of the immune system. Clearly, workload during provisioning does not, on its own, explain the increase in ROMs, because in that case, we would have observed the elevation of ROMs in both the infected and the non-infected group. Likewise, immune activation does not explain ROMs elevations by itself, because in that case, ROMs would have been high in infected birds during all breeding stages. It appears that the elevated ROMs in the infected provisioning birds are associated with a combination of experiencing an increased workload, while also having to deal with the negative (oxidative) effects of activated immune function and impaired oxygen-carrying capacity caused by the infection of red blood cells ([36], but see [31]). Experimental manipulations in which infection intensity in naturally infected birds is reduced through medication, and for which a range of immune and oxidative indices are measured could help us to interpret links between parasitic infection, immune activation and oxidative status (see [34]).

As workload seems to play a role in the extent to which parasitic infection is related to oxidative repercussions, it is likely that higher breeding intensity (i.e. increased brood size) would exacerbate the difficulty of maintaining oxidative balance, particularly in infected birds. In the Seychelles warbler, clutches normally consist of just one egg [52]. In the present dataset, only four out of 46 nests for which original brood size was known contained two offspring, therefore we do not have sufficient power to meaningfully test whether brood size impacted the relationships found. Experimental manipulations of workload would be needed to test this hypothesis (e.g. by brood size manipulations or handicapping provisioning adults, but such experiments would be unfeasible in the endangered Seychelles warbler). A brood size experiment in great tits (*Parus major*) [101] did recently show that—although a direct relationship between malarial infection and oxidative stress resistance was lacking—increased parental effort reduced protection against oxidative stress in males. Moreover, males raising enlarged broods had also higher counts of malarial parasites, indicating that increased breeding intensity can also lead to higher susceptibility to parasites. In our study, it is also possible that provisioning activities led to increased infection intensities in already infected individuals. The higher metabolic demands during this energetically intensive stage could have lowered their ability to resist relapses, thus magnifying the detrimental effects of being infected [28]. Links between breeding activity and malarial relapses (‘spring relapses’) have been documented repeatedly in several other bird species [41,97,102–104].

Finally, it is important to realize that malaria is a complex disease, which complicates the interpretation of study findings. After the initial malaria infection, normally characterized by the most severe physiological and fitness-related consequences [28], birds can enter into a chronic stage with very low infection intensities [28]. When sampling wild birds, it is, therefore, difficult to distinguish whether individuals that test negative for malaria have never been exposed to the parasite, or have cleared the infection. On the other hand, birds that test positive for malaria may either have been caught during the initial acute stage of infection, during a relapse, or may have entered the chronic stage [105]. The best way forward may be to measure infection intensities using quantitative PCR (qPCR) to help make more detailed inferences regarding the disease stages of individual birds and assess whether infection intensities are higher during provisioning. Nevertheless, with our study, we were able to show that parasitic infection is linked with oxidative status, particularly during the provisioning period when energetic demands are high; hence suggesting that higher workload while dealing with an infection can aggravate oxidative repercussions.
